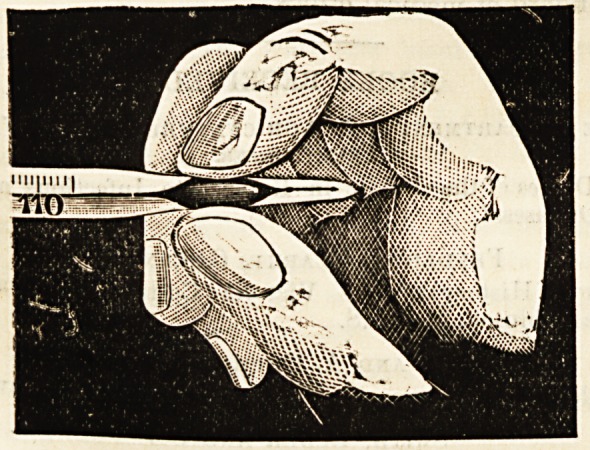# New Appliances and Things Medical

**Published:** 1902-10-04

**Authors:** 


### NEW APPLIANCES AND THINGS MEDICAL.

[We shall be glad to receive at our Office, 28 & 29 Southampton Street, Strand, London, W.C., from the manufacturers, specimens of all new preparations and appliances which may be brought out from time to time.]

### THE REPELLO CLINICAL THERMOMETER.

(G. H. Zeal, 82 Turnmill Street, London, E.C.)

Probably more clinical thermometers are broken in the act of resetting than by any other means. Moreover, some persons appear to find a difficulty in resetting them at all, so that we anticipate a great future for any reliable clinical thermometer which can be reset without having recourse to the violent procedure of "shaking down." The Repello thermometer, which is the invention of Mr. Zeal, appears to us to obviate this difficulty in a very ingenious and practical manner. At the top of the thermometer, which in other respects has no peculiarities, there is a flattened bulb which is filled with mercury, and a small column of dried air, which separates it from the registering column of mercury of the lower bulb. Before using, the thermometer is reset by squeezing the flattened bulb between the thumb and finger: this pressure-action on the small column of air forces the registering column of mercury below the normal point. We have submitted one of these thermometers to practical tests, and find that it fulfils all the claims made for it. It is accurately graduated, reliable, and reset without any trouble.

### DIGESTIVE SAUSAGES.

(William Harris, Harris's Corner, St. John Street, West Smithfield, London, E.C.)

We have examined several samples of Harris's digestive sausages, and find them uniformly of high standard. They are made from meat of the choicest description, and, owing to the special method of preparation adopted by the manufacturer they truly comply with their claim of being digestive. The flavour is excellent, and we know of no sausages which we consider more wholesome or more safe for general consumption.

### TUBERCULO-ALBUMIN (DR. SHANN'S).

(Paul Jahn, 12 Cross Street, Finsbury Pavement, London, E.C.)

Tuberculo-albumin is a clear colourless liquid prepared, according to the statement of the manufacturer, from cultures of tubercle bacilli from which the toxic matter, which causes high temperature and symptoms of poisoning, has been removed. For the sake of keeping the albumin from decomposition 0·3 per cent. of carbolic is added. It is stated that tuberculo-albumin is a specific therapeutic agent in the treatment of tuberculous disease, we need hardly say, however, that its specific character in the treatment of tuberculosis can only be proved by prolonged clinical investigation, and bacteriologically by its influence on the tubercle bacillus *in vitro*.

## Figures and Tables

**Figure f1:**